# Galectin-1-Induced Autophagy Facilitates Cisplatin Resistance of Hepatocellular Carcinoma

**DOI:** 10.1371/journal.pone.0148408

**Published:** 2016-02-09

**Authors:** Yu-Chi Su, Goutham Venkata Naga Davuluri, Cheng-Hao Chen, Dong-Che Shiau, Chien-Chin Chen, Chia-Ling Chen, Yee-Shin Lin, Chih-Peng Chang

**Affiliations:** 1 Department of Microbiology & Immunology, College of Medicine, National Cheng Kung University, Tainan 701, Taiwan; 2 The Institute of Basic Medical Sciences, College of Medicine, National Cheng Kung University, Tainan 701, Taiwan; 3 Department of Pathology, Chia-Yi Christian Hospital, Chiayi 600, Taiwan; 4 Department of Cosmetic Science, Chia Nan University of Pharmacy and Science, Tainan 701, Taiwan; 5 Translational Research Center, Taipei Medical University, Taipei 110, Taiwan; 6 Center of Infectious Disease and Signaling Research, National Cheng Kung University, Tainan 701, Taiwan; Taipei Medical University, TAIWAN

## Abstract

Hepatocellular carcinoma (HCC) is one of the most common cancers in Taiwan. Although chemotherapy is the primary treatment for HCC patients, drug resistance often leads to clinical failure. Galectin-1 is a beta-galactoside binding lectin which is up-regulated in HCC patients and promotes tumor growth by mediating cancer cell adhesion, migration and proliferation, but its role in chemoresistance of HCC is poorly understood. In this study we found that galectin-1 is able to lead to chemoresistance against cisplatin treatment, and subsequent inhibition has reversed the effect of cell death in HCC cells. Moreover, galectin-1 was found to induce autophagic flux in HCC cells. Inhibition of autophagy by inhibitors or knockdown of Atg5 cancels galectin-1-induced cisplatin resistance in HCC cells. Increase of mitophagy triggered by galectin-1 was found to reduce the mitochondrial potential loss and apoptosis induced by cisplatin treatment. Finally, using an in situ hepatoma mouse model, we clearly demonstrated that inhibition of galectin-1 by thiodigalactoside could significantly augment the anti-HCC effect of cisplatin. Taken together, our findings offer a new insight into the chemoresistance galectin-1 causes against cisplatin treatment, and points to a potential approach to improve the efficacy of cisplatin in the treatment of HCC patients.

## Introduction

Diagnosed worldwide, one million people are suffering from liver cancer [1], which ranks the fifth most common cancer, and comes third in cancer-related deaths. Hepatocellular carcinoma (HCC) accounts for around 80–90% of liver cancers. Although a preponderance of cases occurs in Asia and Africa, an upsurge of the mortality rate has been found in North America and Europe [2, 3]. Risk factors such as hepatitis infection, alcohol related cirrhosis, and nonalcoholic fatty liver diseases are considered to influence the increasing the number of HCC cases in both developed countries and low risk areas[4, 5]. Surgical resection and liver transplantation are the first two choices for treatment of HCC patients; however, not all patients are capable of taking surgery or finding a compatible donor. Although treatment with anti-cancer drugs to destroy cancer cells (chemotherapy) can help patients to control cancer growth, unfortunately, liver cancer patients always develop drug resistance to chemotherapy. Although the mechanism of developing chemoresistance is not fully understood, recent evidence has shown that tumor microenvironmental stress-induced autophagy may contribute in part [6].

Autophagy is an evolutionarily conserved self-degradation pathway that could digest the cytoplasmic components via endosome and lysosome fusion resulting in the formation of autophagosomes [7]. Present day research has shown that autophagy plays a critical role in protecting the cancer cell from hypoxia and nutrition deficiency [8, 9]. Moreover, under cellular stress conditions such as radiation and chemotherapy, autophagy is considered to be a potential mechanism that is activated in order to promote the survival of tumor cells. An increasing amount of evidence is unveiling different roles of autophagy in inducing chemoresistance towards the antineoplastic therapies such as cisplatin, doxorubicin and many other drugs[10, 11]. It has been reported that increased autophagy in cancer cells could facilitate their resistance to drug-induced apoptosis [12, 13].How these cancer cells trigger autophagy to tolerate chemotherapy is still unclear.

Lectins are carbohydrate binding proteins which are able to recognize carbohydrates attached to proteins and lipids known as glycoconjugates. One group of this protein family are galectins, which are defined by their propensity in recognizing β-galactose sugar moieties such as laminin, fibronectin, and hensin [14, 15]. Reorganized expressions of galectins seem to be extensively increased in several types of cancer, including HCC[16]. Emerging evidence has clearly shown that galectin-1, especially in the secreted form, is an important member of the galectin family involved in numerous activities including immunosuppression, angiogenesis, metastasis, cell survival and proliferation. Current studies also point out that a marked upsurge in the concentrations of galectin-1 in the blood stream is associated with poor progression-free survival and overall survival in HCC patients [17]. Galectin-1 is known to be a hypoxia regulated protein, and has been suggested as inducing the progression of chemoresistance in epithelial ovarian cancer [18, 19]. However the cancer regulating mechanisms of galectin-1 in inducing chemoresistance are still unclear and a clear understanding of the underlying mechanisms are much needed to improve the efficacy of the chemotherapy treatment in HCC.

In our previous findings we determined the role of a lectin based compound Concanavalin-A (Con A) in the induction of autophagy to treat murine hepatoma [20]. Given the galectin-1 overexpression in HCC and its activity in drug-resistance, we designed this study to investigate the role of soluble galectin-1 in inducing autophagy to provide cisplatin-resistance to the HCC. Our findings demonstrated that blockage of soluble galectin-1 augments the activity of cisplatin both in *vitro* and in *vivo*, suggesting a promising strategy to increase the clinical efficacy of cisplatin to treat highly advanced HCC.

## Results

### Galectin-1 attenuates cisplatin-induced cell death in hepatoma cells

**Fig 1:**
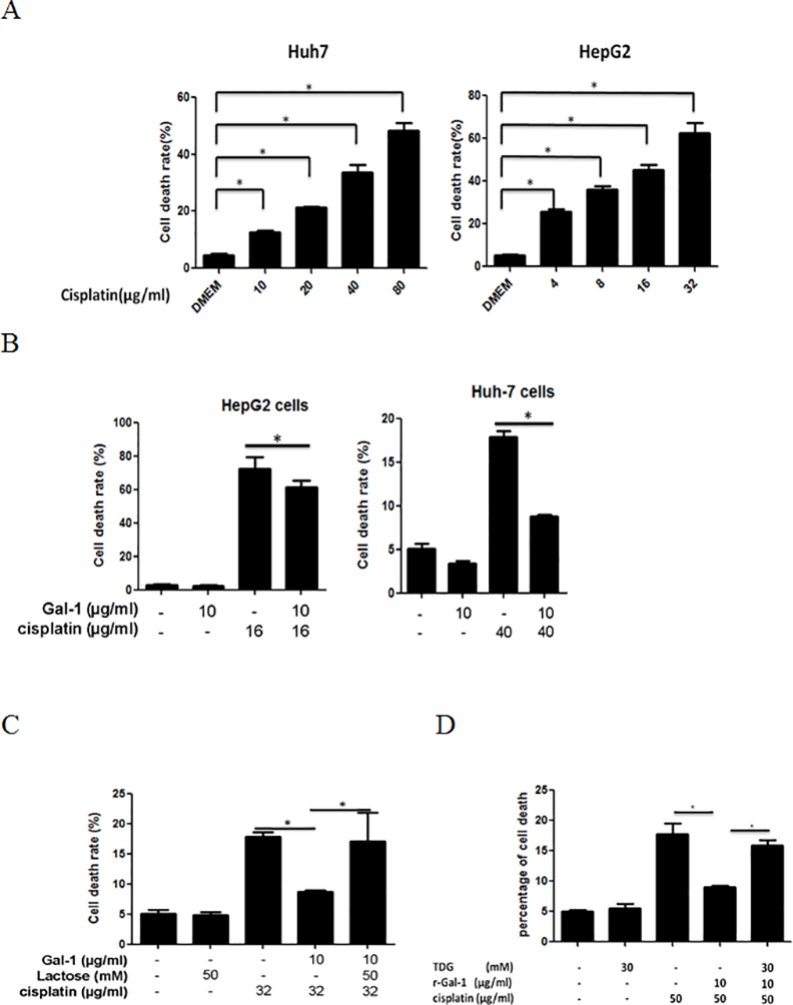
Galectin-1 attenuates cisplatin-induced cell death of hepatoma cells. (A) HepG2 and Huh7 cells were treated with various concentrations of cisplatin for 24 hours. The cell death rate was measured by PI (propidium iodide) staining using flow cytometry. (B) HepG2 and Huh7 cells were pretreated with galectin-1 for 1 hour followed by the treatment with cisplatin for 24 hours. The cell death was determined by PI staining. (C&D) Huh 7 cells were pretreated with respective soluble galectin-1 inhibitors, including β-lactose and TDG, for 1 hour and further treated with cisplatin in the absence or presence of galectin-1 for another 24 hours. The cell death was determined through PI staining. The data shown are the mean ± SEM values of 3 experiments. * p < 0.05.

Galectin-1 is overexpressed in human hepatocellular carcinoma and is accumulated in stroma surrounding tumors [21]. These abundantly secreted galectin-1 proteins are considered to promote tumor cell migration, invasion and metastasis [22]. The role of soluble galectin-1 on chemosensitivity remains unclear. We therefore first tested the effect of soluble galectin-1 on chemotherapeutic agent-treated human hepatoma cells. Since cisplatin is the clinically standardized drug, we employed this drug for our cytotoxic studies. We found that cisplatin at 16 or 40 μg/ml is able to induce around 50–60% cell growth inhibition and death in HepG2 or Huh7 cells, respectively (Fig 1A). Next, we first examined whether the soluble gaectin-1 is able to induce the chemoresistance towards the cisplatin treated hepatoma cells. We pretreated the HepG2 and Huh7 cells with the recombinant galectin-1, either alone or in combination with cisplatin. As shown in Fig 1B, we were able to determine a significant decrease in cell death of hepatoma cells after treating them with recombinant galectin-1. This protective effect of soluble galectin-1 on cisplatin-treated hepatoma cells was abolished in the presence of the galectin-1 inhibitors, lactose or thiodigalactoside (TDG), suggesting that carbohydrate-dependent interaction is responsible for this effect (Fig 1C and 1D). These findings indicate that soluble galectin-1 can induce the chemoresistance towards the cisplatin treatment to hepatoma cells.

### Galectin-1 triggers autophagy via inhibiting AKT/mTOR signaling

**Fig 2:**
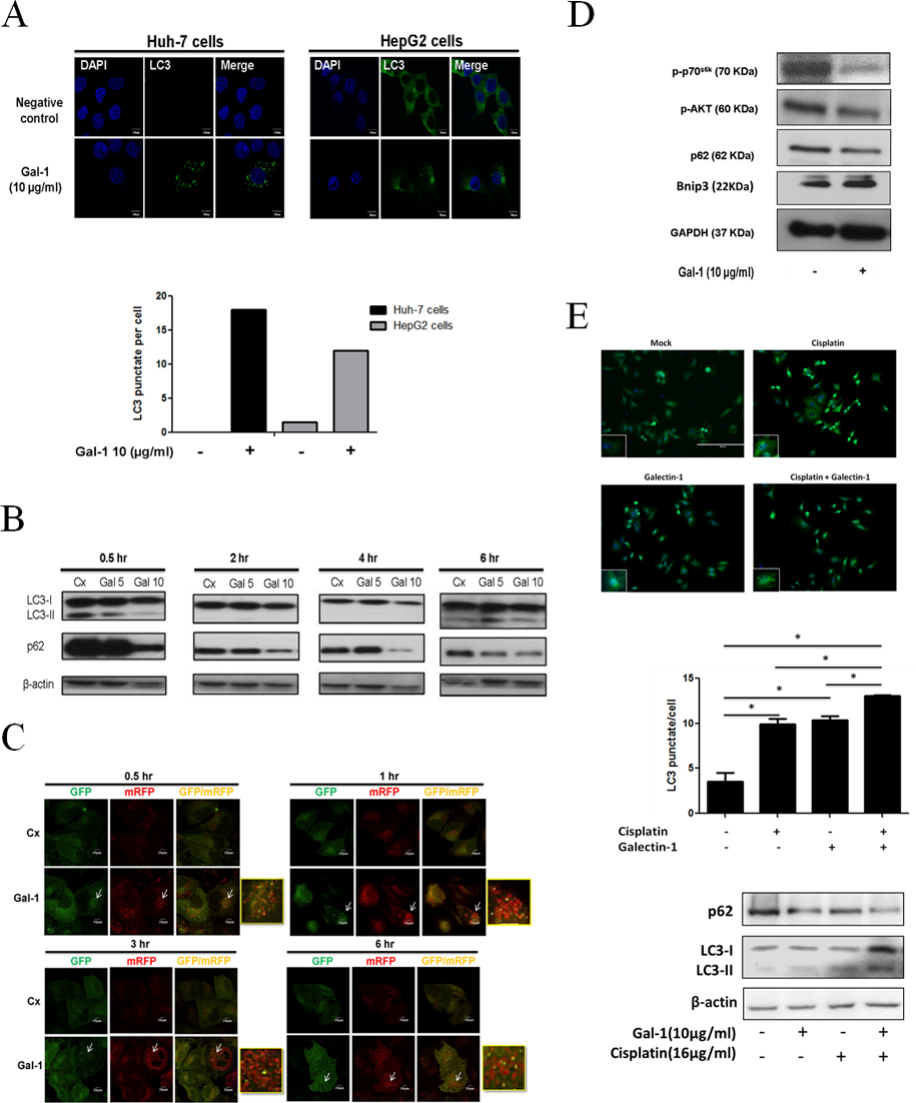
Galectin-1 induces autophagic flux in hepatoma cells. (A) Huh-7 cells and HepG2 cells were treated with galectin-1 (10 μg/ml) for 1 hour and then stained with anti-LC3 antibody. The punctate LC3 was analyzed under a fluorescence confocal microscope. Average numbers of LC3 puncta per cell were quantified. (B) HepG2 cells were treated with galectin-1(5 or 10 μg/ml) for indicated time and then the cell lysates collected. The protein expression of LC3-I/II and p62 was determined by Western blotting. (C) mRFP-GFP-LC3 plasmids were introduced into HepG2 cells by lentiviral vectors and treated with galectin-1 (10 μg/ml) for 0.5, 1, 3 and 6 hours. Cells were then fixed and analyzed under a fluorescence confocal microscope. The white arrows indicate the autophagic flux punctate areas, which are magnified in other representative squares. (D) Huh-7 cells were treated with galectin-1 (10 μg/ml) for 6 hours. The protein expression of p-70^s6k^, p-AKT, p62, and BNIP3 was determined by Western blotting.

Galectin-1 is a carbohydrate binding protein with a diverse set of activities in the human body. In our previous findings [20] we found that Con A, a mannose-binding lectin can induce autophagy in hepatoma cells. We therefore investigated whether exogenous soluble galactin-1 could show a similar activity in inducing autophagy in hepatocellular carcinoma. As shown in Fig 2A, significant inductions of LC3 accumulation were detected by recombinant galactin-1 in both HepG2 and Huh-7 cells. The galectin-1-triggered LC3-II conversion and p62 degradation could be detected as early as 30 minutes after treatment (Fig 2B). To further confirm the galectin-1 induced autophagic flux, cells were transfected with mRFP-GFP tandem fluorescent-tagged LC3 (tfLC3) and then treated with galectin-1. When autophagosomes are fused with lysosomes, GFP-LC3 will be degraded by acid-activated lysosomal enzymes, but not mRFP-LC3 [23]. As shown in Fig 2C, tf-LC3 transfected HepG2 cells treated with galectin-1 showed an upsurge of mRFP-LC3 punctate within 6hrs of treatment, indicating that soluble galectin-1 triggers an autophagic flux in hepatoma cells. Since galectin-1 is found to induce autophagic flux, we further investigated the regulating pathways that are involved in the induction of autophagy. According to our earlier findings, an autophagy inducing factor BNIP3 is responsible for Con A-induced autophagy. Hence we investigated whether soluble galectin-1 is able to induce the up-regulation of BNIP3 in hepatoma cells. It was found that galectin-1 is able to induce a significant increase in BNIP3, whereas both p-AKT and p70s6k showed a significant down-regulation (Fig 2D). This indicates that galectin-1 is able to suppress the mTORC signaling and activate BNIP3 to induce autophagy. These results demonstrated that exogenous galectin-1 is capable of inducing autophagic flux in hepatoma cells by upregulating the BNIP3 and decreasing the mTORC signaling.

### Galectin-1 mediated autophagy facilitates cisplatin resistance in hepatoma cells

**Fig 3:**
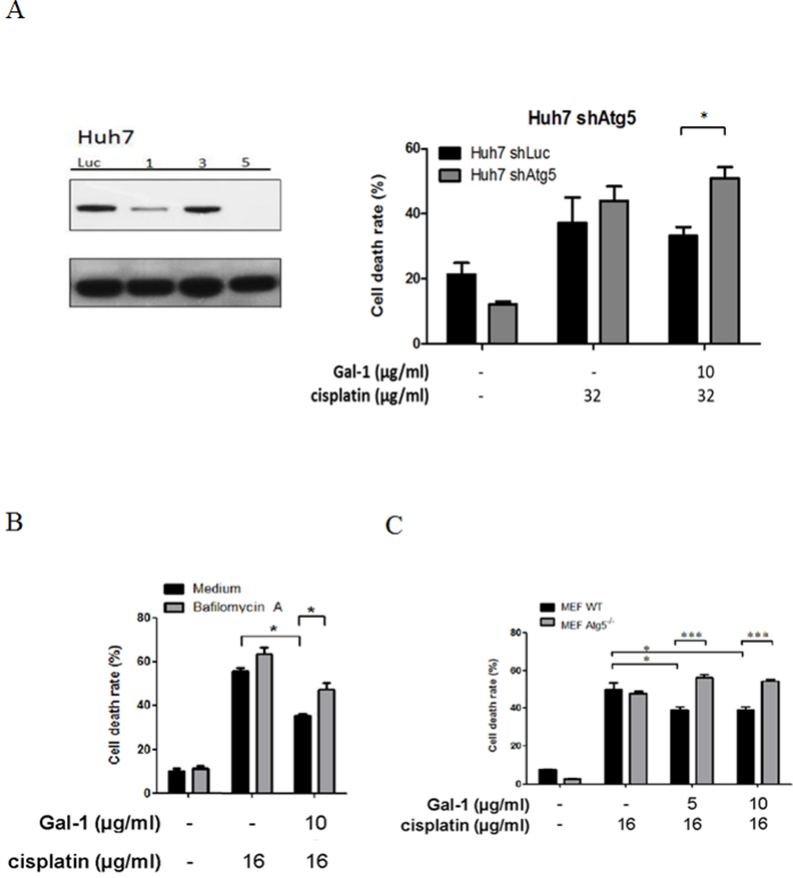
Autophagy contributes to galectin-1-induced cisplatin resistance of hepatoma cells. (A) Atg5 protein was knocked down in Huh-7 cells by shRNA (clone 5). Huh-7-shLuc and Huh-7-shAtg5, cells were pretreated with galectin-1 (10 μg/ml) for 1 hour, and then further treated with cisplatin for another 24 hours. The cell death was determined by PI staining. (B) HepG2 cells were pretreated with autophagy inhibitor bafilomycin A1 (25 nM) for 1 hour and then treated with galactin-1 (10 μg/ml) and cisplatin. After 24 hours post treatment, the cell death was determined by PI staining. (C) Wild-type (WT) and Atg5-/- cells were pretreated with galectin-1 (10 μg/ml) for 1 hour and then further treated with cisplatin (16 μg/ml) for another 24 hours. The cell death rate was measured by PI staining. * p < 0.05; ***p<0.01.

From the previous findings it is well established that autophagy is a key mechanism to induce chemoresistance in cancer cells. Galectin-1 overexpression has been indicated to promote progression and chemoresistance towards cisplatin in epithelial ovarian cancer [18]. Next we further investigated the significance of galectin-1-induced autophagy to provide the resistance to cisplatin. Hence, in order to determine the role of galectin-1 we generated galectin-1 knockdown cells using a lentiviral vector containing shRNA of Atg5. Subsequently, wild type or Atg5 knock down hepatoma cells were pretreated with or without galectin-1, and then with cisplatin, after which their chemoresistance was evaluated. According to the data shown in Fig 3A, a remarkable increase in cell death was observed in Atg5 silencing cells compared to wild-type cells. The resistance to cisplatin -induced cell death triggered by galectin-1 was also reversed in the presence of autophagic inhibitor bafilomycin-A1 (Fig 3B). Furthermore, to confirm whether autophagy is crucial for galectin-1-induced cell protection, we treated wild type or autophagy-deficient Atg5^-/-^ MEF cells with cisplatin in the presence or absence of galectin-1.Consistent with the above findings, an increase of cisplatin-induced cell death was observed in galectin-1-pretreated Atg5^-/-^ MEF cells compared to wild type cells (Fig 3C). These results indicate that autophagy is upregulated by galectin-1, and thus facilitates the chemoresistance in hepatoma cells.

### Galectin-1 triggers mitophagy to attenuate cisplatin-induced apoptosis

**Fig 4:**
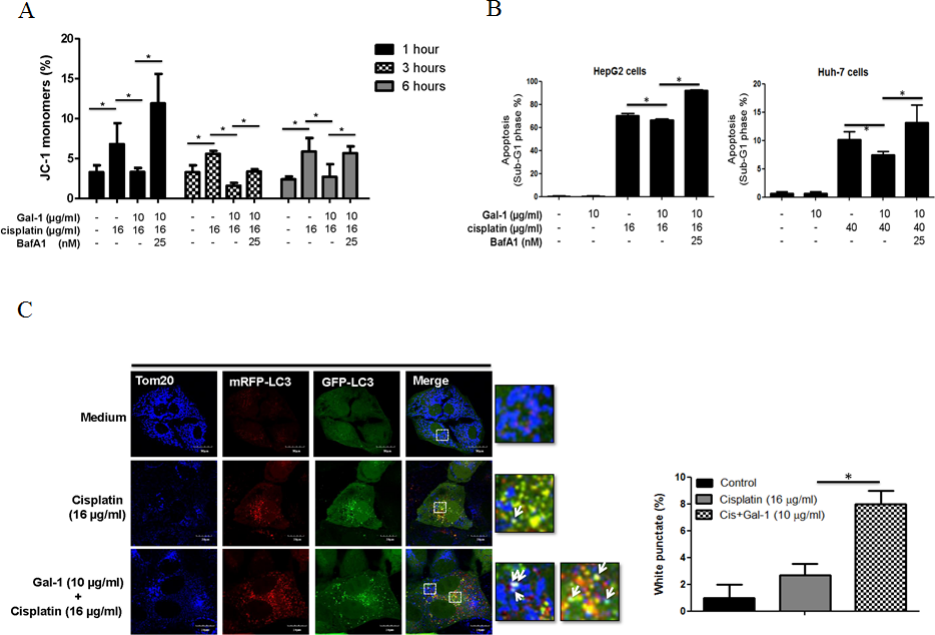
Galectin-1 enhances mitophagy to reduce cisplatin-triggered apoptosis. (A) HepG2 cells were pretreated with autophagy inhibitor bafilomycin A1 (25 nM) for 1 hour, and then further treated with galectin-1 (10 μg/ml) and cisplatin for the indicated time. The depolarized mitochondrial membrane percentage was measured by JC-1 staining and quantified. (B) Huh-7 and HepG2 cells followed above experiment treatment. The cell apoptosis (sub-G1 phase %) was measured by PI staining after 70% alcohol fixation at -20°C overnight. (C) mRFP-GFP-LC3 plasmids were introduced into HepG2 cells by lentiviral vectors and treated with galectin-1 (10 μg/ml) for 1 hour and then treated with cisplatin (16 μg/ml) for another 3 hours. Cells were then fixed and stained with anti-Tom20 antibody. The protein distribution was analyzed under the confocal microscope. White arrows indicate the targeted mitochondria by autophagosome. The quantification results were calculated from 3 individual experiments.* p < 0.05.

Cisplatin promotes cancer cell apoptosis by inducing mitochondrial potential loss and damage. In the presence of autophagy, these damaged mitochondria are removed by autophagosomes, which is known mitophagy, and hence cells are protected from harm or destruction, and their viability is sustained [24, 25]. Accordingly, since the above results show that galectin-1 can induce autophagy, we further investigated whether this autophagy can protect the mitochondrial potential loss induced by cisplatin. Therefore, the potentiometric mitochondrial dye JC-1 was used to quantify the depolarization. JC-1 forms complexes with intense red fluorescence in healthy cells, while it remains in monomeric form with green fluorescence in apoptotic cells which contain low mitochondrial potential. As predicted, cisplatin-treated cells showed an increased formation of JC-1 monomers and apoptosis, indicating a mitochondria-mediated cell death. Interestingly, in the presence of galectin-1, cisplatin-triggered potential loss and apoptosis were both attenuated in hepatoma cells. This galectin-1-triggered protection was reversed by autophagic inhibitor bafilomycin A1 (Fig 4A and 4B), suggesting that galectin-1 reduces cisplatin-induced mitochondrial damage via autophagy. Since induction of mitophagy may facilitate the elimination of damaged mitochondria, we next explored whether galectin-1 triggers mitophagy. To further confirm this, HepG2 cells were transfected with tf-LC3 which were then treated with cisplatin, with or without galectin-1. Using Tom-20 staining as an indication of mitochondria, galectin-1 in presence of cisplatin was found to induce fusion of autophagosomes and mitochondria in a significant increase compared to cisplatin-treated or control group (Fig 4C). In summary, from this data we found that galectin-1 induces mitophagy during the cisplatin treatment and decreases the membrane potential loss, and also protects the cells from undergoing apoptosis.

### Galectin-1 inhibition increases the antitumor activity of cisplatin in hepatoma-bearing mice

**Fig 5:**
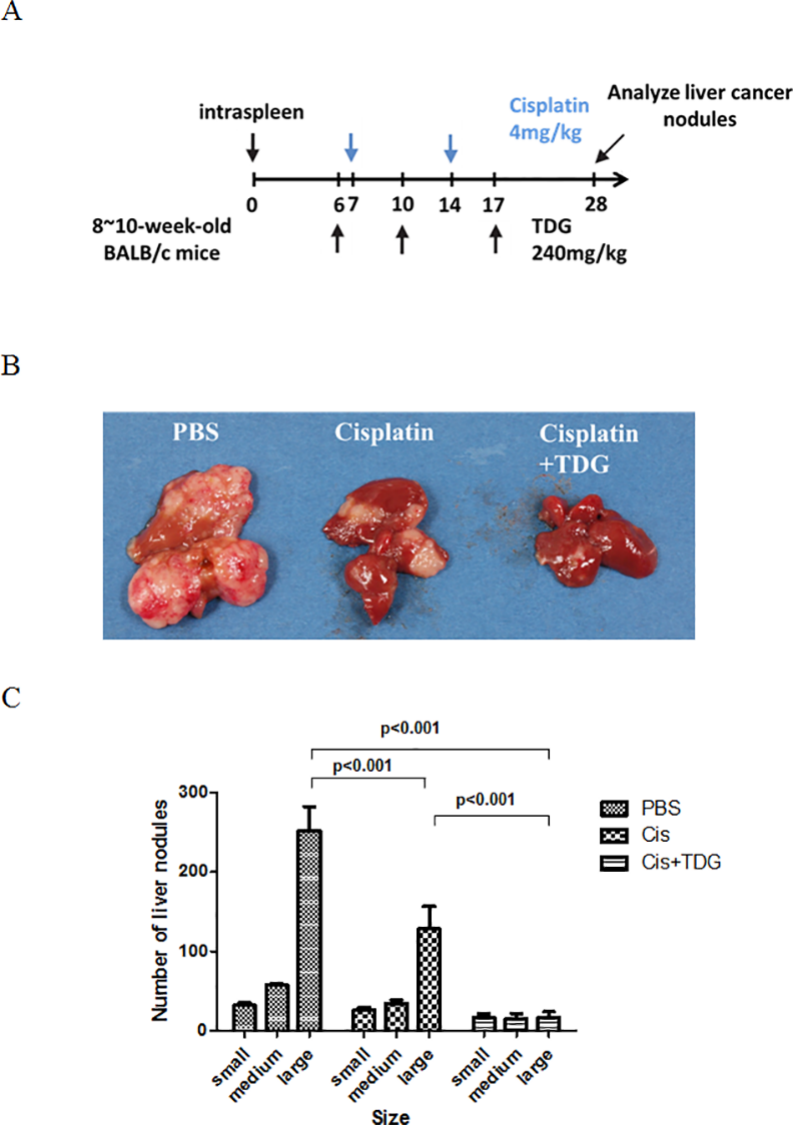
Galectin-1 inhibitor increases anticancer activity of cisplatin in hepatoma-bearing mice. (A) 8–10 week old BABL/C mice were intrasplenically inoculated with mouse hepatoma cells ML-1_4a_ to establish tumor nodule formation (n = 8). Cisplatin (5mg/kg) was given to mice twice at a 7- day interval beginning on day 7. TDG (240mg/kg) was given to mice on day 6, 10 and 17. The number and sizes of the tumor nodules in the liver were determined on day 28. (B-C) After 28 days of treatment, mice were sacrificed and the livers were sectioned to analyze the number and size of live nodules. Results were quantified from three independent experiments.

To further determine the anti-tumor benefit of inhibiting the galectin-1 in combination with cisplatin, we used an *in situ* mouse hepatoma model generated by intrasplenic grafting of autologous hepatoma cells, ML-1 cells [26]. The tumor cells were first colonized in the spleen, and then migrated to the liver to form visualized nodules around 5 to 7 days post inoculation. All the mice survived until the end of the study with no alterations of general toxicity and body temperature. The anti-tumor activity of cisplatin given intraperitoneally was investigated. After 6 days post tumor cell inoculation, mice were randomly assigned to pretreatment with TDG (240mg/kg) every 4 days with or without cisplatin (5mg/kg)(Fig 5A). Upon the finalization of treatment all the mice were sacrificed and their livers were removed and the cancer nodules were analyzed. The results are shown in Fig 5B and 5C, revealing that the number of liver nodules was reduced in cisplatin-treated hepatoma-bearing mice compared to control mice. Moreover, this anti-tumor activity of cisplatin was further enhanced in combination with TDG. Our findings therefore suggest that inhibition of galectin-1 can increase the anti-cancer activity of cisplatin.

## Discussion

**Fig 6:**
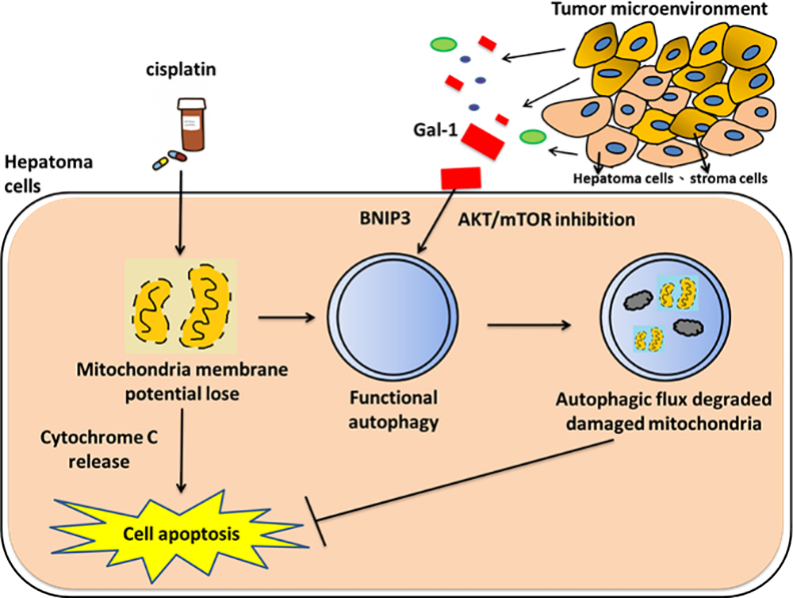
Model of galectin-1-induced autophagy triggers cisplatin resistance. In hepatoma microenvironment, cisplatin can stimulate hepatoma cells to undergo mitochondria-mediated apoptosis. However, free form galectin-1 secreted by stroma cells or hepatoma cells can bind to hepatoma cells and trigger autophagic flux via inhibiting AKT-mTOR activities. Galectin-1-induced autophagy can target cisplatin- damaged mitochondria to reduce both mitochondrial membrane potential loss and cell death, thus providing a chemoresistance to cancer cells.

HCC is one of the most leading cancers worldwide. Although the intra-arterial infusion of combinational chemotherapy is considered to be the predominant method to control the metastatic disease, the survival ratio is very low due to the chemoresistance often induced towards the treatment. Intensifying the chemosensitivity towards the drugs is an essential task in improving the potential of drugs in clinical use. In our study we found that exogenous galectin-1 is able to induce chemoresistance to cisplatin in hepatoma cells. This chemoresistance induced by soluble galectin-1 is facilitated through the induction of BNIP3-related autophagy and also by downregulating the mTORC signaling. Atg5-/- knockdown hepatoma cells were also manifested to be less effective in exhibiting the chemoresistance towards the cisplatin treatment. We have also identified that galectin-1 stimulates the autophagy process to remove damaged mitochondria induced by cisplatin treatment. Furthermore, galectin-1 inhibitor TDG can increase the anti-tumor activity of cisplatin to hepatoma-bearing mice (Fig 6). These findings add a new deep understanding of the pathogenic roles of galectin-1 in hepatoma cells.

Hepatic arterial infusion of cisplatin is widely used worldwide to treat the highly advanced HCC [27]. Even though there is a high responsiveness towards the drug activity in the beginning, eventually the cells start developing resistance towards these drugs. Emerging research has demonstrated that galectin-1 overexpression can lead to chemoresistance towards cisplatin in epithelial ovarian cancer [18]. Similar to the above approach, Le Mercier, M., et al have shown that reduced galectin-1 expression could induce the increased sensitivity towards the pro-apoptotic and pro-autophagic drugs in glioma. More recently it was shown that galecitn-1 is capable of increasing the expression of p38 MAPK, ERK and COX-2 proteins to induce chemoresistance and progression of lung cancer cells [28]. These studies determined that endogenous galectin-1 can induce numerous factors which account for the chemoresistance in cancer. However the effect of soluble galectin-1 in providing the chemoresistance is not yet clearly determined. In this study we have demonstrated that hepatocellular carcinoma cells were able to resist the treatment of cisplatin in the presence of soluble galectin-1. We have also found that the soluble galectin-1 inhibitors such as lactose and TDG were successfully able to sensitize the hepatocellular carcinoma cells towards the cisplatin treatment. This demonstrates that the increase in the level of soluble galectin-1 can provide the chemoresistacnce to hepatocellular carcinoma towards the cisplatin treatment which confirms the role of soluble galectin-1 as similar to the above findings of endogenous galectin-1.

Recent studies have shown that autophagy is a mechanism that reduces the sensitivity of cancer cells towards the chemotherapeutic agents by decreasing the drugs’ apoptotic potential. Even though the molecular mechanisms in regulating autophagy are not yet clear, autophagy has become a major target to inhibit the chemoresistance in cancer cells. In our previous studies we have revealed that Concanavalin-A, a member of the lectin binding family, can induce autophagy in hepatoma cells [20]. Galectin-8, a predominant member of galectin family is found to induce NDP52-mediated autophagy to restrict *Salmonella* proliferation [29]. Following the above studies, here we further demonstrated that soluble galectin-1 induces autophagy in hepatocellular carcinoma cells in response to chemotherapeutic drugs. This suggests that mammalian lectin-induced autophagy may regulate various biological functions which merit further investigation. It has been shown that several signaling pathways are involved in the regulation and maintenance of the autophagy level inside the cells. The mTOR signaling pathway is one such regulating pathway that plays a crucial role in suppressing the activity of autophagy. In this study we observed that there is a significant down-regulation of autophagy suppressing proteins such as p70s6k and p-AKT. Moreover, we have also demonstrated that soluble galetin-1 increased the expression of beclin-1 binding protein BNIP3 in the progression of autophagy. These results suggest that soluble galectin-1 is able to induce beclin-1 mediated autophagic flux and also down-regulates the mTORC signaling pathway in response to the chemotherapeutic treatment. However, the signaling mechanisms have to be studied in detail.

Earlier research has suggested that the release of cytochrome c from mitochondria during apoptotic cell death is through the opening of the mitochondrial permeability followed by swelling and rupture of the mitochondrial outer membrane [30]. Loss of mitochondrial membrane potential is usually found on treatment with chemotherapy drugs. It has been also shown that cisplatin-based chemotherapies are known to induce apoptosis by reducing the mitochondrial potential in ovarian cancer cells[31]. On the other hand, cancer cells may develop strategies to prevent drug-induced mitochondrial damage, such as autophagy. Recent research has shown that an increase of autophagy activity can attenuate apoptosis and mitochondrial potential loss induced by cisplatin in hepatoma cells. Inhibition of autophagy in cisplatin treated ovarian cancer cells has amplified a great response of apoptosis through a mitochondrial dependent pathway [32]. These studies have suggested that autophagy is able to inhibit the mitochondrial potential loss induced by cisplatin to provide chemoresistance towards several types of cancer. Several possible mechanisms of anti-apoptosis activities of autophagy have been raised. Removing damaged mitochondria by autophagy is one kind of protective mechanism for cells to counter cytochrome c-mediated apoptosis. In this present study, we did find increased mitophogy via immunostaining by galectin-1 in cisplatin-treated hepatoma cells, suggesting that galectin-1-triggered autophagy may help cells to eliminate damage mitochondria and resist chemotherapy drugs. However, how galectin-1 increases mitophagy in cisplatin-treated cells is not clear. Beclin-binding protein BNIP3 has been implicated to target mitochondria for autophagosome degradation under hypoxia stress [33]. Significant up-regulation of BNIP3 was induced by galectin-1 in hepatoma cells (Fig 2D). Hence, we speculated that BNIP3 is involved in glectin-1-triggered mitophagy.

Recently it has been shown that depletion of galectin-1 can extend the life of melanoma bearing mice [34]. In this study we further reported that galectin-1 can induce autophagy to antagonize cisplatin-caused hepatoma cell death, and the enhanced antitumor effect was seen in BALB/c mice, with the cisplatin treatment in combination with TDG being associated with inhibition of soluble galectin-1. However, the appropriate use of TDG and cisplatin as anti-tumor drugs depends on the dose and frequency of drug administration. Cisplatin has been previously reported to be nephro-cytotoxic, being associated with increased renal vascular resistance and histologic damage to proximal tubular cells at a concentration of 10mg/kg [35]. However, at the dose of 5mg/kg in this study, no nephro-cytotoxity is observed in hepatoma-bearing mice. Systemic administration of cisplatin *in vivo* reduced the number of tumor nodules in tumor bearing mice compared to the control. Furthermore, combined treatment with TDG and cisplatin has decreased the tumor nodules in a significant ratio and regained the liver weight compared to cisplatin alone *in vivo* (data not shown). These findings illustrate the importance of soluble galectin-1 in tumors, and the efficacy of combined treatment with galectin-1 inhibitor and cisplatin for co-operatively reducing the chemoresistance of hepatoma.

In our study we have shown that soluble galectin-1 could induce autophagy in response to the cisplatin treatment. This suggests that high levels of galectin-1 in the hepatoma microenvironment during the cisplatin treatment could facilitate the chemoresistance of cancer cells. Thus our findings have uncovered a new role of galectin-1 in promoting the chemoresistance in hepatocellular carcinoma, and so represent a promising approach to vanquish the chemoresistance acquired towards the cisplatin treatment.

## Materials and Methods

### Reagents and Antibodies

The chemicals cisplatin, bafilomycin A1, thiodigalactoside and β-Lactose were purchased from Sigma-Aldrich (MO, USA). Recombinant human galactin-1 was purchased from R&D systems Inc. (MN, USA). The JC-1 dye for mitochondrial membrane potential was purchased from Thermo Fisher Scientific Inc. (NY, USA). Plasmid mCherry-EGFP-LC3B was kindly provided by Dr. Tamotsu Yoshimori (Osaka University, Japan). For primary antibodies, those against Tom 20 were purchased from Santa Cruz Technology Inc. (CA, USA), those against Atg5, pospho-AKT 1/2/3, AKT and phospho- p70s6kinase were from Cell Signaling Technology (MA, USA), those against microtubule-associated proteins light chain 3 (LC3) and anti-p62/ sequestosome1 were from MBL (Nagoya, Japan), that against Bcl-2/adenovirus E1B 19 kDa-interacting protein 3 (BNIP3) was from Sigma-Aldrich, that against β-actin was from Abcam (MA, USA), and that against GAPDH was from Ambion (TX, USA).

### Cell culture and immunofluorescent staining

Human hepatoma cell lines, Huh7 and HepG2, were obtained from the Cell Collection and Research Center (CCRC, Hsin-Chu, Taiwan). BALB/c hepatoma cell line ML-1_4a_ cells were adapted from ML-1 cells in BALB/c mice for four generations as previously described [20]. Atg5-/- mouse embryonic fibroblasts were kindly provided by Dr. Tamotsu Yoshimori (Osaka University, Japan). Cells were cultured in DMEM (Gibco, Grand Island, NY) supplemented with 10% FBS, L-glutamine and penicillin-streptomycin. For immunofluorescence staining, cells were fixed with 4% paraformaldehyde in PBS for 10 mins and permeabilized with 0.1% Triton-x-100 for another 15 minutes. Then cells were washed and stained with anti- LC3 antibody, followed by secondary antibody conjugated with Alexa Fluor 488. The LC3 punctation was observed under a confocal fluorescence microscope (Olympus FV 1000, Japan).

### Cell death and apoptosis assay

Huh-7 and HepG2 were seeded into a 12 well plate and treated with cisplatin or recombinant galectin for 24 hours. Subsequently, cells were collected and stained with propidium iodide (PI, 10 μg/ml) for 30 minutes. The cell death was further determined by detecting PI-positive stained cells using flow cytometry (BD FACSCalibur™, USA). To detect sub-G1 apoptotic cell population, the cells were first fixed with 70% cold ethanol at 4°C overnight and then stained with PI. The sub-G1 apoptotic cell population was determined and analyzed by flow cytometry.

### Western blot

The cells were treated with desired concentrations of drugs and collected to homogenize in cell lysis buffer (20 mM Tris-HCl pH 7.5, 150 mM NaCl, 1 mM Na2EDTA, 1 mM EGTA, 1% Triton, 2.5 mM sodium pyrophosphate, 1 mM beta-glycerophosphate, 1 mM Na3VO4, 1 μg/ml leupeptin). Proteins were separated through SDS gel electrophoresis and transferred onto the PVDF membranes. The PVDF membranes were blocked with 5% skimmed milk and incubated with the appropriate desired primary antibodies at 4°C. Later on the membranes were washed and incubated with peroxidase-conjugated secondary antibodies. The blots were visualized by enhancing chemiluminescence reagents (PerkinElimer Life Sciences, Boston, MA).

### Lentivirus-based short hairpin RNA (shRNA) transfection

Atg5 was silenced in hepatoma cells by stably expressing the lentivirus based shRNA targeting the human Atg5. The clone was obtained from National RNAi Core Facility (Institute of Molecular Biology/Genomic Research Center, Academia Sinica, Taiwan). The following oligonucleotides were used for the shRNA experiments: Atg5: TRCN0000151963 5’–CCTGAACAGAATCATCCTTAA-3’, and that for control luciferase is TRCN0000072247 5′-GAATCGTCGTATGCAGTGAAA-3′. To generate the recombinant lentivirus, two helper vectors, pCMVdeltaR8.91 and pMD.G, and a target vector PLKO.1-puro-shRNA were transfected to 293T cells. Hepatoma cells were infected with the recombinant lentivirus for 48 hours and puromycin was employed to select the stably expressed cells. The knockdown efficiency of shRNA was further confirmed by Western blotting as described above.

### JC-1 staining

Treated cells were washed twice with PBS and further dissolved. JC-1 dye was added to each sample with a final concentration of 2 μM and incubated at 37°C for 30 minutes. Cells were then washed twice with PBS and resuspended back in PBS for flow cytometry analysis.

### Mouse in situ hepatoma model

BALB/c mice (male, 8–10 weeks old) were purchased from the Animal Laboratory of National Cheng Kung University (Tainan, Taiwan). All mice were maintained in the pathogen-free facility of the Animal Laboratory of National Cheng Kung University. The animals were raised and cared for according to the guidelines set up by Institutional Animal Care and Use Committee (IACUC) of National Cheng Kung University. This study was approved by the Committee on the Ethics of Animal Experiments of National Cheng Kung University (Permit Number: 102117). All mice were anesthetized by sodium pentobarbital (50mg/kg via intraperitoneal injection) before the surgery, and administered with meperidine (4 mg/kg via intraperitoneal injection) after surgery to minimize suffering of mice. A murine in situ hepatoma model was set up by intrasplenic injection of 1x10^6^ viable ML-1_4a_ cells into anesthetized mice as previously described[26]. After 6 days post tumor inoculation, the mice were assigned to treatment with cisplatin alone or in combination with TDG. Cisplatin was administered intraperitoneally at 5mg/kg on day 7 and 14 in the course of treatment. TDG (240mg/kg) was pretreated 1 day before the cisplatin treatment via intraperitoneal administration. The clinical signs, including body weight loss and lethargy, of hepatoma-bearing mice were monitored every two days after tumor inoculation. There were no significant clinical signs of mice found during all experiments. All mice were alive and healthy during the time of sacrifice. Mice were sacrificed after 28 days of tumor inoculation by CO_2_ asphyxiation. The maximum size of liver nodules that were allowed to grow before euthanizing the mice was 5mm in diameter. The tumor was weighed and subjected for the calculation of number of tumor nodules.

### Statistical Analysis

Statistical analysis was performed by using Graphpad Prism software. Statistical comparisons of the experimental data between the treatment and control groups were made using the analysis of student’s t-test or one-way analysis of variance (ANOVA) followed by Tukey’s multiple-comparison posttest. The experimental results were expressed as the means ± SD significant differences. p<0.05 is considered to be statistically significant.
